# Adaptive blood pressure–modulated atrial pacing in hypertensive HFpEF patients: a randomized, first-in-human study

**DOI:** 10.1093/eschf/xvaf020

**Published:** 2026-01-19

**Authors:** Michael H Burnam, Santosh Kumar Sinha, Mukesh Jitendra Jha, Sanjay Kumar Sharma, Amitesh Nagarwal, Rohit Chopra, Eli S Gang

**Affiliations:** BaroPace, Inc., Issaquah, WA, USA; L.P.S Institute of Cardiology & Cardiac Surgery, GSVM Medical College, Swaroop Nagar, Kanpur, Uttar Pradesh, India; L.P.S Institute of Cardiology & Cardiac Surgery, GSVM Medical College, Swaroop Nagar, Kanpur, Uttar Pradesh, India; Cardiology Department, Maharaja Agrasen Superspeciality Hospital, Jaipur, India; Cardiology Department, NIMS Medical College, NIMS University, Jaipur, India; Cardiology Department, C M Chopra Hospital & Heart Care Centre, Chomu, India; BaroPace, Inc., Issaquah, WA, USA; Cardiovascular Research Foundation, Beverly Hills & Cedars-Sinai Smidt Heart Institute, Los Angeles, CA, USA

**Keywords:** Hypertension, HFpEF, Rate adaptive atrial pacing, Exercise tolerance, Chronotropic incompetence, Cardiac Pacing

## Abstract

**Introduction:**

Heart failure with preserved ejection fraction (HFpEF) represents approximately 50% of all heart failure cases and lacks effective treatments. Chronotropic incompetence contributes to exercise intolerance in these patients. This study evaluated the safety and efficacy of blood pressure–adaptive atrial pacing (BPAP) vs standard bradycardia pacing (STD) in hypertensive patients with HFpEF.

**Methods:**

In this prospective, double-blind, randomized, self-controlled crossover study, 16 patients (mean age: 62.7 ± 10.9 years; 6% female; left ventricular ejection fraction 55.3 ± 3.8%) with treated hypertension and implanted dual-chamber pacemakers underwent two 3-week treatment phases (BPAP and STD) in random order. The BPAP algorithm–modulated atrial pacing rate in response to home blood pressure readings. Endpoints included the Minnesota Living With Heart Failure (MLWHF) score, New York Heart Association (NYHA) class, 6-minute walk test (6MWT), and modified Bruce treadmill test.

**Results:**

BPAP improved MLWHF score by an additional 15% from baseline (*P* = .0288), whereas STD showed a non-significant 3% worsening. Exercise time increased significantly during BPAP (+83.2 ± 55.6 s, *P* = .005) but not during STD (+70.8 ± 84.4 s, *P* = .095). The 6MWT distance rose by 35.8 ± 29.9 m during BPAP (*P* = .003) vs minimal change with STD (+8.2 ± 40.1 m, *P* = .6). NYHA class improved in 55.6% of BPAP patients vs 11% with STD (*P* = .0455). Mean heart rate was higher during BPAP (83.8 ± 8.3 bpm) than STD (72.9 ± 12.0 bpm, *P* < .0001), with no difference in systolic blood pressure (137.5 ± 14.9 vs 138.6 ± 14.0 mmHg, *P* = .68). No adverse events occurred.

**Conclusion:**

In hypertensive patients with HFpEF and implanted pacemakers, BPAP safely improved exercise capacity and functional status compared to standard pacing. The approach demonstrates feasibility of home-based blood pressure–modulated pacing for physiologic rate adaptation. (NCT06036186)

## Graphical Abstract

**Graphical Abstract:**
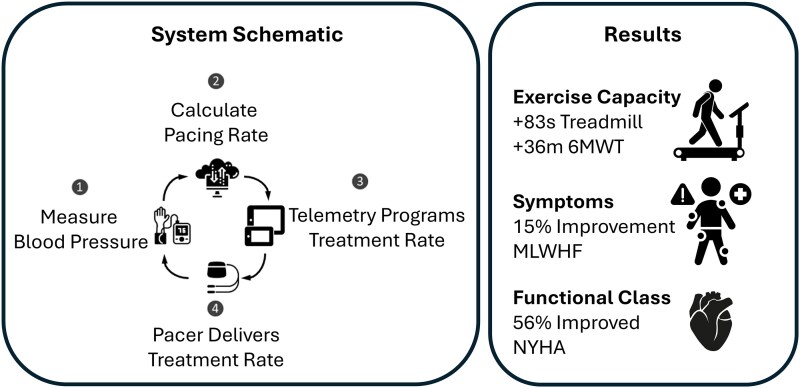
This randomized, double-blind, crossover study evaluated blood pressure-adaptive atrial pacing (BPAP) in hypertensive HFpEF patients with implanted pacemakers. BPAP dynamically adjusted paced atrial rate in response to twice-daily blood pressure measurements. Compared with standard pacing, BPAP improved exercise capacity [+83 s treadmill time; +36 m six-minute walk test (6MWT)], reduced symptoms (Minnesota Living with Heart Failure Score of -15%), and improved functional class (NYHA, 56%). These findings support BPAP as a feasible, physiologic, and well-tolerated rate-adaptation strategy.

## Introduction

Heart failure patients with preserved ejection fraction (HFpEF) comprise half of all patients with heart failure.^1^ Patients with HFpEF present with exercise intolerance and a blunted heart rate (HR) response to exercise. Chronotropic incompetence is present in over 50% of these patients.^2–4^

Despite the prevalence of HFpEF, available therapies are limited. While therapies to lower HR, typically via pharmacological means, which promote relaxation and enhance filling of the ventricles, have shown benefits for heart failure patients with reduced ejection fraction (HFrEF), there is limited evidence to support this in HFpEF patients.^5–7^ To the contrary, lower HR has been associated with adverse outcomes in this patient population;^7^ nonetheless, despite a lack of evidence supporting β-blocker therapy in HFpEF patients, they are frequently prescribed in daily clinical practice.^8^ Indeed, to test the hypothesis that increasing HR may improve clinical outcomes in HFpEF patients, attempts at increasing pacing rates in such patients have been recently published.^9^  ^,10^

We report the results of a prospective, double-blind, self-controlled, randomized, crossover study evaluating the efficacy and safety of a novel atrial pacing algorithm in treating patients suffering from HFpEF associated with hypertension (HTN). The algorithm modulates right atrial (RA) pacing rate in response to blood pressure cuff measurements (BPAP) performed at the patient’s home.

## Methods

### Trial design and oversight

This prospective, double-blind, self-controlled, randomized, crossover study evaluated the feasibility, efficacy, and safety of the PressurePace™ system (BaroPace, Issaquah, WA) in treating patients suffering from Heart Failure with preserved Ejection Fraction (HFpEF) associated with hypertension. The PressurePace algorithm modulates right atrial pacing rate in response to blood pressure.

This study was conducted at four clinical sites in Kanpur, Jaipur, and Chomu, India. The Medical Device Advisory Committee and Central Drugs Standard Control Organization (CDSCO) under Directorate General of Health Services, Ministry of Health & Family Welfare approved this study. The study was prospectively registered with ClinicalTrials.gov (NCT06036186).

### Trial participants

Patients were enrolled in the study after meeting all inclusion and no exclusion criteria. Major inclusion criteria include prior implantation of a Pacetronix (Pithampur, India) dual-chamber pacemaker, age between 35 and 90 years, diagnosis of heart failure with preserved ejection fraction (>45%), systolic blood pressure (SBP) > 135 mmHg despite at least one medication for the treatment of hypertension, able to tolerate ≥ 3 min of a modified Bruce protocol treadmill test, physically capable of performing 6-minute walk tests with a minimum distance of ≥225 m, stable medications for hypertension for at least 4 weeks prior to screening. Additionally, right atrial pacing < 50% and average intrinsic/pacing heart rate < 70 bpm over the previous clinical monitoring period of the pacemaker diagnostics of at least 14 days. Major exclusion criteria include a resting SBP of >170 mmHg, DBP of >120 mmHg, end-stage renal disease on haemodialysis, prone to atrial or ventricular arrhythmias with altered pacing, taking short-acting Nifedipine or Clonidine, or taking beta blockers with intrinsic sympathomimetic activity (ISA).

All participants provided written informed consent prior to participation in any study-specific activity.

### PressurePace system

**Figure 1:**
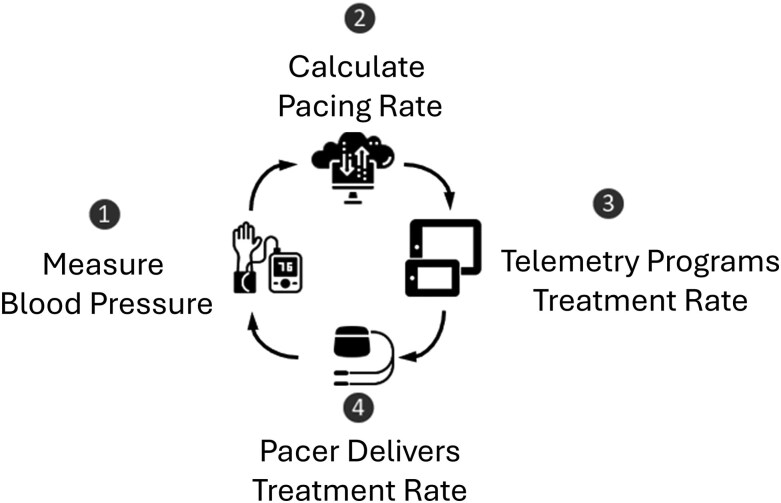
PressurePace system schematic. The system is comprised of a tablet device connected to a wireless blood pressure cuff. The tablet was able to connect to the pacemaker to control the atrial pacing rate. Each blood pressure measurement was wirelessly sent to the tablet, where the PressurePace algorithm could modify the programmed atrial pacing rate of the pacemaker. The PressurePace algorithm then decided to either increase, decrease, or leave the atrial pacing rate unchanged

The PressurePace system consists of a tablet device containing PressurePace software and connected to a wireless blood pressure cuff (*Figure 1*). The tablet is connected to the pacemaker to control the atrial pacing rate. Patients would take a blood pressure measurement 12 h apart each day, which was transmitted to the tablet where the PressurePace algorithm could modify the programmed atrial pacing rate of the pacemaker. The PressurePace algorithm analysed the difference between the patient's systolic blood pressure (BP) and a target BP. Based on this difference, the algorithm then decided to either increase, decrease, or leave the atrial pacing rate unchanged. The maximum change in pacing rate allowed by the algorithm was 10% from the prior pacing rate, not to exceed 88 bpm.

### Trial procedures

**Figure 2:**
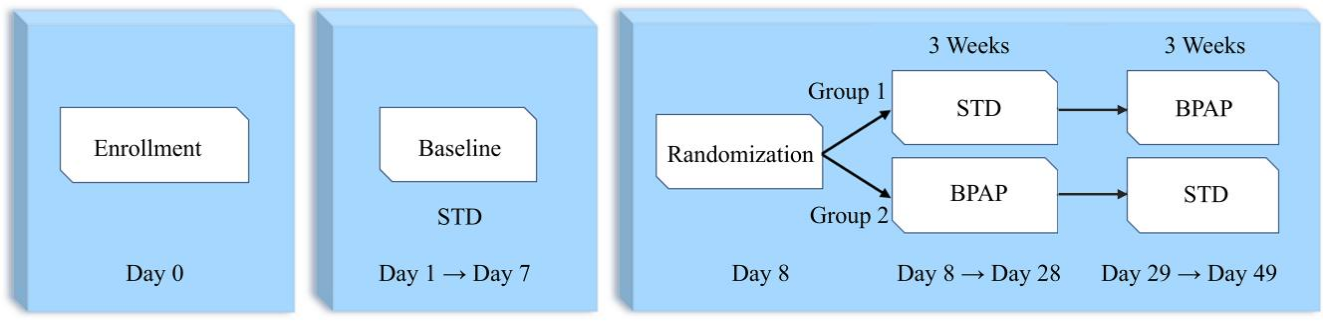
Trial overview. The trial was broken into three parts. Screening/Baseline, (Days 0–7) followed by randomization and two 3-week treatment regimens, either standard bradycardia pacing (STD) or right atrial pacing rate modulated in response to blood pressure cuff measurements (BPAP)

The trial was broken into three parts. Screening/Baseline, followed by randomization into either Group 1 or Group 2 to undergo two treatment regimens (*Figure 2*). Screened patients that met all inclusion and no exclusion criteria entered a stabilization phase (Days 1–7) where patients continued to receive antihypertensive medications with pacemakers programmed to standard bradycardia pacing (STD). Patients underwent testing identical to that occurring post-randomization, including Minnesota Living With Heart Failure (MLWHF) Questionnaire, New York Heart Association (NYHA) classification, 6-minute walk test (6MWT), modified Bruce protocol treadmill exercise test, and blood pressure measurements. On Day 7, testing was repeated, if a patient met any of the exclusion criteria or following conditions: >3 lbs. weight loss, >10 mmHg drop in SBP, or >5 mmHg drop in DBP (mean of the past 3 12-h BP readings at home), the patient was screen-failed from the study. Eligible patients were then randomized to a treatment group. Group 1 (STD-to-BPAP) underwent STD for the first 3 weeks (Days 8–28) followed by 3 weeks of BPAP treatment (Days 29–49), and Group 2 (BPAP-to-STD) underwent BPAP treatment followed by STD (Days 29–49). While undergoing BPAP pacemaker programming was modified twice a day to adjust the right atrial pacing rate in response to systolic blood pressure >135 mmHg. Systolic and diastolic blood pressure (SBP and DBP) were measured via two methods throughout the baseline period and each treatment arm post-randomization. First, an ambulatory blood pressure cuff-measured BP 27 times over 24 h at baseline and the end of each treatment regime (BPAP and STD). Second, two daily measurements were taken by the patient approximately 12 h apart using the study-provided BaroPace system. The BP measurement was used by the PressurePace algorithm to determine if the atrial pacing rate should be increased, decreased, or left unchanged. Trained clinic staff were present at the patient’s home during these measurements to monitor the process and would only interact if there was an error reading or the cuff placement was incorrect.

Immediately prior to each morning (AM)/evening (PM) BP measurement, patients were asked if they felt well enough in the prior 12 hto proceed, and a Kardioscreen EKG was obtained, which was overread by blinded cardiologists to rule out atrial fibrillation. Blood pressure was then measured and wirelessly transmitted to the PressurePace algorithm, which determined if the atrial pacing rate needed to be changed and if so, should it be increased or decreased. Three minutes post BP measurement, regardless of the treatment regimen the patient was in, the tablet asked if the patient still felt well. If the patient responded yes, the session ended, and any changes were saved by the pacemaker. If the patient responded ‘No, I feel worse’ and was undergoing BaroPacing, the algorithm would reduce the atrial pacing rate change by 50%, wait 3 min, and repeat the question. If after the second prompt the patient felt well, then the settings were saved to the pacemaker. If the patient indicated they still felt bad, and they felt ok during the prior 12 h, then the pacemaker settings reverted to the settings at the beginning of the session.

At the end of each treatment regimen (either STD or BPAP) the MLWHF, NYHA classification, 6MWT, and modified Bruce protocol treadmill exercise test were repeated. Total study duration was approximately 7 weeks and patients’ pacemakers were returned to pre-study programming at study exit.

### Outcomes and assessment

The objective of this study was to evaluate the safety and effect of BPAP on exercise tolerance and heart failure symptoms in patients with HFpEF associated by hypertension compared to STD.

The primary trial end points were two-fold: first to show safety via the absence of related adverse and serious adverse events, and second, to show the effect of blood pressure–modulated pacing on heart failure symptoms as assessed by MLWHF questionnaire and on exercise tolerance as measured by a modified Bruce protocol treadmill test. Secondary endpoints included changes in NYHA classification and 6MWT compared to baseline.

### Randomization and blinding

A BaroPace system was issued to every patient who reached randomization (Day 7). The PressurePace system interface included a random number generator, which randomized to either Group 1 (STD-to-BPAP) or Group 2 (BPAP-to-STD). Randomization was blinded to the patient, physician, and clinic staff. Each patient visit occurred in the same fashion with blood pressure measurements being collected by the PressurePace System and then a programming step where the device rate was modified if the patient was receiving BPAP, or where the device programming was left unchanged if the patient was receiving STD treatment. The steps involved in each programming session appeared exactly the same to all involved parties regardless of treatment regime.

### Statistical analysis

The sample size was typical for feasibility studies and not based on statistical considerations. Study group homogeneity at baseline was determined by performing two-sample *t*-tests or a Fisher’s exact test to compare the patients’ characteristics and outcome measures.

A mixed model was initially planned to analyse the relationship between the effect of STD (control) and that of BPAP (therapy) across two groups, assuming no learning or carryover effect. However, post-study data suggested the presence of both learning and carryover effects. To address this, for quantitative endpoints, the linear mixed model was adjusted to evaluate treatment effects within each group (STD-to-BPAP and BPAP-to-STD). The treatment effect within each group was estimated from the treatment coefficient, and the differences in improvement from Week 3 to Week 6 between groups were assessed using a two-sample *t*-test. For qualitative endpoints, Fisher’s exact test or χ2 tests were applied to compare the response rate of BPAP vs the response rate of the STD treatment regimen per group.

Results are presented as a mean ± SD, and *P* < .05 was considered significant. Statistical analyses were performed using R Statistical Software (v4.4.2).

## Results

### Trial population

**Figure 3:**
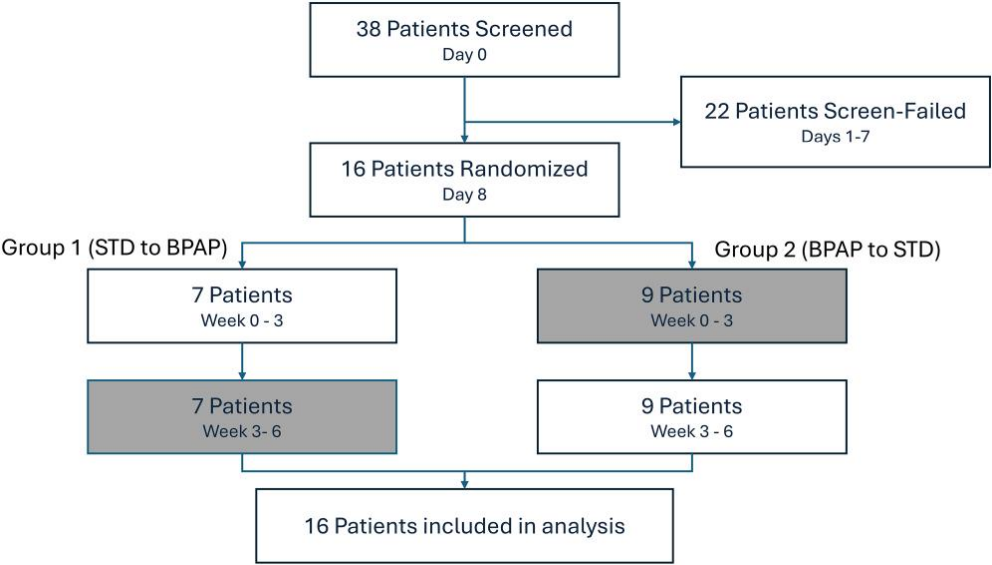
Patient disposition. A total of 38 patients were screened for the trial, with 16 enrolled and randomized. The shaded blocks represent the BPAP treatment regimen. Group 1 underwent STD in the first treatment regimen, followed by BPAP for the second treatment regimen, while Group 2 underwent BPAP and then STD

From August 2023 to February 2024, a total of 38 consecutive patients were screened for inclusion in the study. Of those 38 patients, 22 were screen-failed prior to randomization as shown in *Figure 3*. The remaining 16 patients were randomized to either treatment Group 1, which underwent BPAP for the first 3 weeks followed by 3 weeks of STD, or Group 2, which underwent STD for the first 3 weeks followed by 3 weeks of BPAP.

The baseline characteristics of the patients are listed in *Table 1*. The mean age was 62.7 ± 10.9 years, and 94% (15/16) of patients were male. All patients were NYHA class II (56%) or III (44%) with an average left ventricular ejection fraction of 55.3 ± 3.8%, and SBP/DBP of 139.3 ± 10.8/85.4 ± 9.0 mmHg. Primary indications for pacemaker implantation were due to advanced conduction system disease (75%), and significant Sinus node disease (25%). Baseline per cent pacing in the RA and RV was 22% and 95% respectively.

**Table 1:** Baseline patient characteristics

Parameter	Value (*N* = 16)
**Age, years**	62.7 ± 10.9
**Gender**	
**Male**	15/16 (94%)
**Female**	1/16 (6%)
**Primary Indication for device**	
**Advanced conduction system disease**	12/16 (75%)
**Significant sinus node disease**	4/16 (25%)
**Device Programming**	
**DDD**	100%
**Cardiovascular**	
**SBP, mmHg**	139.3 ± 10.8
**DBP, mmHg**	85.4 ± 9.0
**LVEF, %**	55.3 ± 3.8
**NYHA**	
**I**	0/16 (0%)
**II**	9/16 (56%)
**III**	7/16 (44%)
**IV**	0/16 (0%)
**Pacing Metrics**	
**RA Pacing, %**	21.7 ± 18.4
**RV Pacing, %**	94.5 ± 9.3

Values are mean ± SD, *n*/*N* (%).

SBP, systolic blood pressure; DBP, diastolic blood pressure; LVEF, left ventricular ejection fraction; NYHA, New York Heart Association; RA, right atrial; and RV, right ventricular.

### Primary outcomes

The results and data analysis are displayed using a designation of two groups:

Group 1: Patients randomized to STD during the first 3 weeks, and subsequently crossed over to BPAP; Group 2: patients randomized to initially undergo BPAP pacing, then crossed over to STD. *P* Values comparing Weeks 3–Week 0, and Weeks 6–Week 3 are given for both groups.

**Figure 4:**
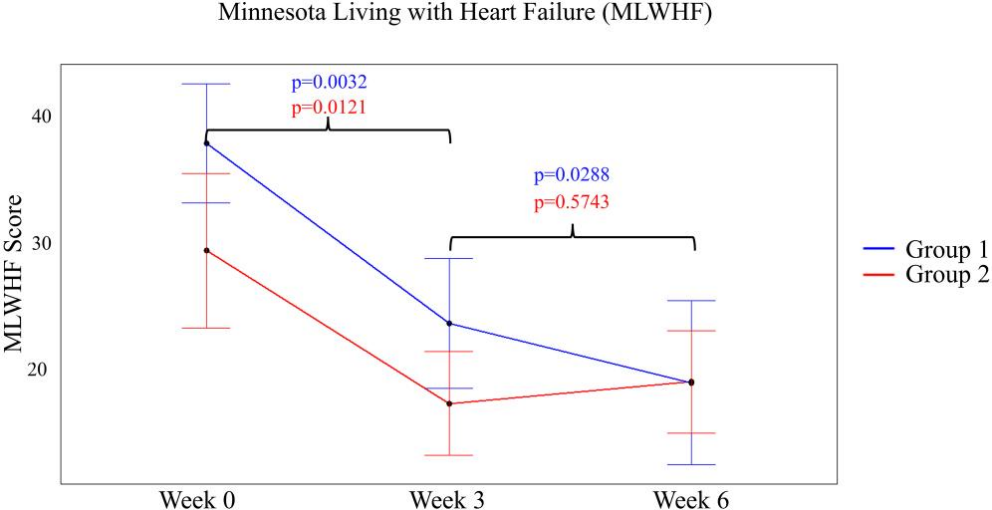
Minnesota Living with Heart Failure (MLWHF). MLWHF Score from Baseline (Week 0) through the first and second treatment regimen (Weeks 3 and 6, respectively) for Groups 1 and 2

**Figure 5:**
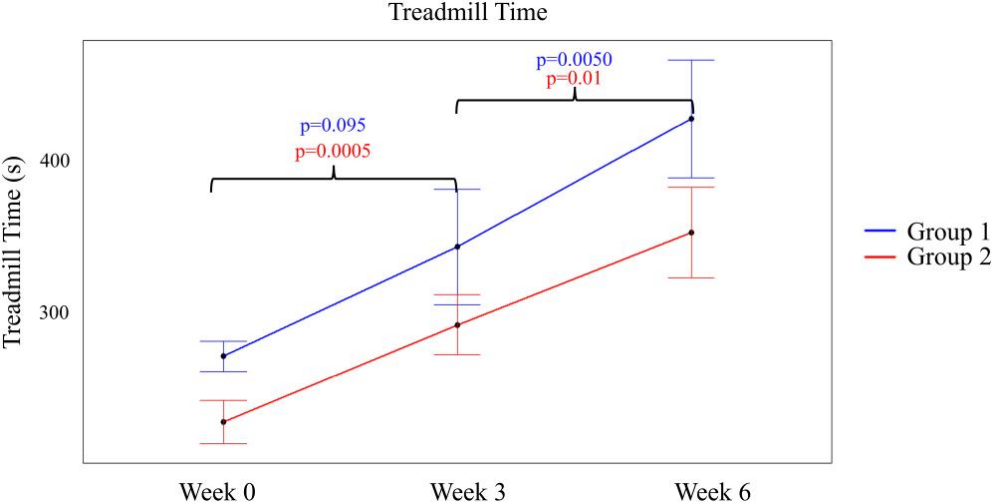
Treadmill time. Treadmill Exercise Times in (seconds) at Baseline (Week 0) and the first and second treatment regimen (Weeks 3 and 6, respectively) for Groups 1 and 2

The effects of BPAP on heart failure symptoms and exercise tolerance were assessed via the MLWHF Questionnaire (*Figure 4*) and exercise time measured during a modified Bruce protocol treadmill test (*Figure 5*). *Figure 4* shows the MLWHF score at Baseline, Week 3, and Week 6 for both Groups 1 and 2. At baseline, the two groups did not significantly differ. A statistically significant improvement was seen in MLWHF scores from Baseline to Week 3, regardless of pacing mode (*P* = .0032, 0.0121). For Group 1, turning on BPAP (Weeks 3–6) resulted in an additional 15% improvement of MLWHF scores (*P* = .0288). In contrast, Group 2 undergoing STD resulted in an insignificant worsening of 3% in MLWHF scores (*P* = .57).

*Figure 5* shows the treadmill exercise time at Baseline, Week 3, and Week 6 for both Groups 1 and 2. Despite randomization, the two groups were statistically different at baseline (*P* = .0422). In Group 1, patients undergoing STD had an increase of 70.8 ± 84.4 s (*P* = .095) (Baseline to Week 3). BPAP resulted in a highly significant, incremental increase of 83.2 ± 55.64 s (*P* = .0050).

In Group 2, patients undergoing BPAP had an increase of 63.3 ± 33.9 s (*P* = .0005) (Baseline to Week 3). Turning off BPAP resulted in a further insignificant increase (*P* = .0777). While exercise time increased in both treatment regimens, BPAP resulted in a significant increase, while STD did not.

### Secondary outcomes

**Figure 6:**
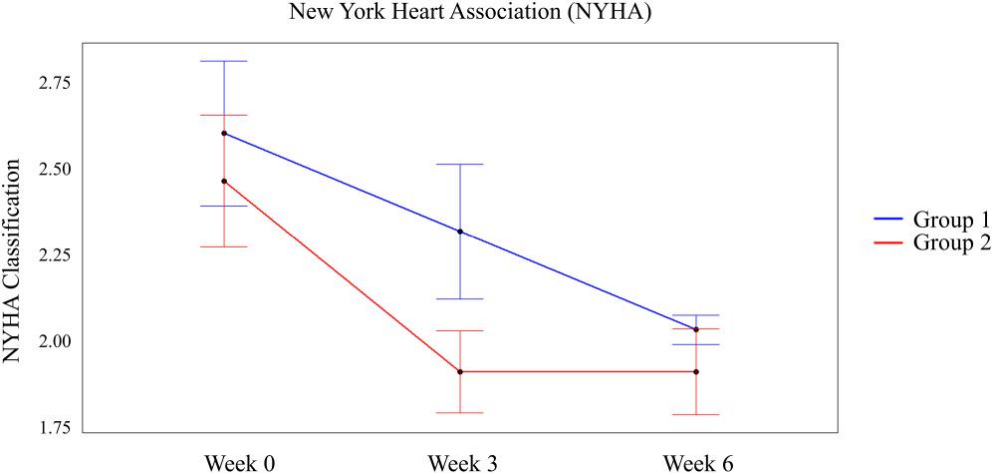
New York Heart Association (NYHA) class. Average numerical values of NYHA scores at Baseline (Week 0) and the first and second treatment regimens (Weeks 3 and 6, respectively) for Groups 1 and 2

**Figure 7:**
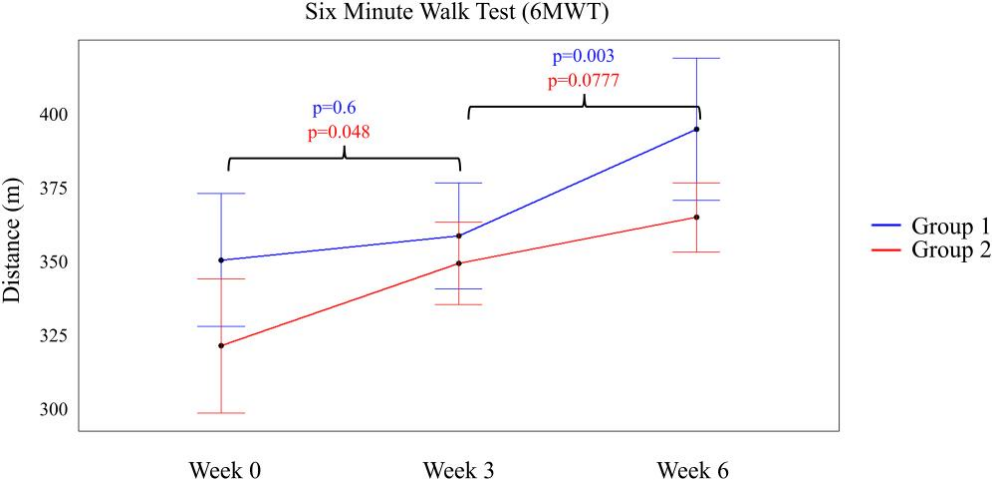
Six-minute walk test (6MWT), distance in metres at baseline (Week 0) and the first and second treatment regimens (Weeks 3 and 6, respectively) for Groups 1 and 2

Secondary end points included NYHA class (*Figure 6*) and 6MWT (*Figure 7*). As shown in *Figure 6*, Group 1 reported improvements in NYHA during STD pacing and continued to improve during subsequent BPAP. In contrast, among Group 2, BPAP improved NYHA at a greater rate than STD during the first 3 weeks, with no further improvement upon crossing over to STD. The rate of improvement in NYHA for during BPAP was 55.6% (*P* = .0455 χ2), compared to 11% for STD.

Changes in exercise capacity were also measured by 6MWT. Data for the 6MWT were available for 15 of 16 patients. *Figure 7* shows the distance walked for Groups 1 and 2 at baseline, and at the end of each treatment regimen. In Group 1, 6MWT improved slightly (8.2 ± 40.09 m) while undergoing STD (*P* = .6). After BPAP, an additional increase of 35.8 ± 29.86 m was observed (*P* = .003). In Group 2, a difference of 27.8 ± 35.63 m while undergoing BPAP with an additional increase of 15.4 ± 19.84 m (*P* = .0777) after STD was observed.

### Cardiovascular effects

Heart rate, SBP, and DBP were measured twice daily throughout the study. The average HR for patients receiving BPAP was 83.8 ± 8.3 compared to 72.9 ± 12.0 bpm (*P* < .0001) for STD. There was no statistically significant difference in SBP regardless of pacing therapy 137.5 ± 14.9 vs 138.6 ± 14.0 mm Hg, BPAP or STD, respectively. Comparing DBP measurements for patients undergoing BPAP vs STD, a statistically significant increase of 2.9 mm Hg was found (*P* = .041) with DBP of 86.6 ± 10.59 mm Hg while undergoing BPAP vs 83.7 ± 7.6 mm Hg while undergoing STD. Twice daily blood pressure measurements showed a narrowing of pulse pressure of 3.19 for BPAP compared to a widening of 3.89 mm Hg while undergoing STD treatment regime (*P* = .0448).

Analysis of pulse pressure using the 24-h measurements showed a narrowing of pulse pressure by 4.2 when undergoing BPAP vs a widening of 6.77 mm Hg (*P* = .0254) during the STD treatment regime. The change in pulse pressure was due to an increase in DBP (74.68 ± 9.98 during STD vs 80.16 ± 9.3 mm Hg during BPAP). Comparing SBP, for both STD and BPAP, it remained unchanged (131.47 ± 15.43 during STD vs 131.71 ± 15.41 mm Hg during BPAP).

Further analysis of pulse pressure via a linear regression, with pulse pressure as the covariate and change from baseline as the response, revealed patients with narrower pulse pressure tended to walk longer on a treadmill (*P* = .0762), walk longer distances in the 6-minute walk test (*P* = .0374), and have better MLHFQ scores (*P*-value = .035) compared to those with wider pulse pressure.

### Interventions

Of potentially 1346 visits, 26 visits 26/1346 (1.9%) were not completed due to patient availability. A total of 1320 completed visits where blood pressure was taken occurred. Of the 1320 visits, 638 and 682 occurred in the STD and BPAP treatment regimens, respectively. In the STD treatment phase 43% (273/638) of the time, the BPAP algorithm, while not active, would have changed the atrial pacing rate. Similarly, in the BPAP treatment phase, the algorithm recommended a change in pacing rate 45% (306/682) of the time. Of the 306 opportunities to adjust the pacing rate, a change was made in 18% (55/306). In the remaining cases, the HR was at the upper limit of the algorithm’s pacing range (88 bpm); thus, the pacing rate was not adjusted. When the algorithm suggested a change in pacing rate, an increase was indicated 76% (42/55) of the time while a decrease was indicated during the remaining 24% (13/55).

### Safety

No adverse events were reported in either arm with either treatment modality.

## Discussion

The authors submit that the important findings reported in this study are as follows:

- To our knowledge, this is the first study in which a cuff-measured blood pressure, measured in a patient’s home, was used to modulate atrial pacing rate of a permanently implanted pacemaker system; hence a closed-loop system utilizing home blood pressure measurement for dictating pacing rate has been demonstrated to be feasible and readily adaptable by the patient.
- The safety of measuring blood pressure at home and transmitting the result via a simple tablet has been demonstrated. At each of the 1320 patient-encounters, the patient had the option of reporting discomfort with the change, and no such events occurred during the study.
- In the population studied, i.e. older patients with HFpEF, treated hypertension, and pre-existing permanent pacemakers, pacing with BPAP resulted in enhanced subjective (MLWHF, NYHA) scores, as well as objective measured performance parameters (treadmill exercise time and 6MWT).

The authors are cognizant of the fact that in each of the measured endpoints, an improvement was measured after 3 weeks, irrespective of pacing group assignment. We attribute this uniform finding to a ‘learning effect’ in which the patients became comfortable with the physical nature of the activity tests (treadmill or 6MWT) and with completion of subjective functional assessment questions (NYHA and MLWHF). Of importance, however, was the statistically significant *continued* improvement when BPAP was present vs *absent or minimal* improvement when the conventional pacing arm was the second arm of the study.

The physiological basis of this improvement remains to be definitively elucidated, but it is known that elevation of left ventricular filling pressure both at rest and with exercise is common in patients with HFpEF.^10,11^ Hence, pharmacologic interventions with GLP-1 agonists, SGLT-2 inhibitors, and a trial of persistent accelerated pacing at rest have shown modest clinical improvements, apparently by reducing LV filling pressures.^9^ As summarized recently, continuous pacing at moderately higher rates favourably alters the end-diastolic pressure-volume curve and thereby reduces left-sided pressure.^9^ Infeld *et al*.^9^ found that moderately accelerated pacing at rest was clinically beneficial for patients with improved MLWHF, NT-proBNP levels, activity levels, and device-detected atrial fibrillation compared to pacing at 60 bpm. Conflicting results, on the other hand, were reported by Reddy *et al*. (RAPID-HF)^10^ who investigated the effects of sensor-driven increased heart rates in patients with HFpEF and chronotropic incompetence and showed that increasing exercise heart rate in these patients *failed* to improve exercise capacity and was associated with *increased* adverse events.^9,10^ Kitzman *et al*.^12^ explained this negative study by hypothesizing that the increase in paced exercise heart rate was counterbalanced by a reduction in stroke volume, thus leaving cardiac output unchanged.

In contrast to these two published studies of pacing in patients with HFpEF, our study was designed to dynamically offer pacing alterations in response to a physiologic parameter, BP, and to lessen or minimize the pacing rate changes when BP criteria were not met. This closed-loop approach seeks to mimic the body’s normal physiologic behaviour in which alterations in function (such as heart rate) are in *response* to changing physiologic conditions. Our group has previously reported that patients with drug-resistant hypertension show a significant lowering of systolic and diastolic blood pressure, which correlated with the amount of atrial pacing.^13^ We have also recently reported improved exercise tolerance with BPAP among patients with HFpEF and hypertension undergoing randomized modified treadmill tests.^14^ We are mindful of the conclusions of the large clinical trial in HFpEF patients showing a worse clinical outcome amongst those with a SBP <130^15^ and have therefore designed the BPAP system to attempt to maintain SBP at an ‘optimal’ range, insofar as possible within the vicissitudes of daily life. This can only be done with a physiological input such as SBP that allows dynamic regulation of pacing changes. In contrast to other pacing studies in the HFpEF population, this algorithm recommended *decreasing* the pacing rate 24% of the time, thus further indicating that a single increase in the pacing rate may not be a physiologic and effective pacing strategy.

The cardiovascular effects of BPAP in this study can be summarized as follows: (a) the average HR during BPAP was significantly higher than during STD, which, of course, would be expected given the pacing algorithm that was being evaluated; (b) the effect of BPAP on SBP was not significant in this patient population, but (c) the DBP tended to increase with BPAP, as measured twice per day as well as the 24-hour blood pressure cuff measurements. The net effect was, therefore, a higher paced HR and a narrower pulse pressure provided by BPAP pacing. In this study, a significant correlation between narrowing of pulse pressure and longer distance walked in the 6-minute walk test, as well as improved MLHFQ scores and a trend towards prolongation in treadmill time, was seen. Indeed, the clinical improvement seen in BPAP-paced patients in this study is in keeping with previous observations in drug-treated patients with HFpEF, wherein reductions in pulse pressure were associated with a lower risk of cardiovascular events, a finding that is in contrast to other forms of heart failure.^16^ Finally, it should also be pointed out that in order to meet the study endpoints, relatively few alterations in atrial pacing rates were required during the 3 weeks of BPAP (average of 18%, maximum of 100%). It may be that neurohumoral effects are triggered by relatively minimal changes in HR and that these effects may have a relatively long duration of effects, i.e. a sort of neurohumoral ‘remodelling’, a phenomenon that has previously been described.^17^ The results of this study potentially highlight the need for a closed-loop system based on physiological parameters, such as BP, that can serve as a proxy for cardiac demand. Further study is warranted to better characterize the effects of BPAP and elucidate the underlying mechanisms.

## Study limitations

Limitations of the study include the small sample size, the relatively short duration of each treatment regime (3 weeks), which may have emphasized the initial ‘learning effect’ and not provided enough time for revealing the magnitude of the differences between treatment regimes. Additionally, during the initial 1-week run-in period, 50% of patients’ blood pressure stabilized. Due to practice habits amongst the investigating physicians, no patients had been programmed to activity-driven pacing. As such, activity-driven pacing was not assessed in this study. Given the negative results of prior studies using activity-driven exercise pacing in HFpEF patients^9,10^ this was not considered a significant issue by our group. Additionally, factors affected patient selection, such as diagnosis of HTN and exclusion of atrial fibrillation. Suzuki *et al.*  ^16^ showed that a lower sBP in patients with HFpEF was associated with worse long-term clinical outcomes. Accordingly, we chose to enrol HFpEF patients with HTN who could be brought to the systolic BP ‘sweet spot’ with BP-responsive atrial pacing without risking excessive lowering of the BP in this population, who are frequently also receiving multiple vasoactive medications as such, an inclusion criteria of sBP > 135 was implemented. NT-Pro-B-type natriuretic peptide (BNP) was not collected during this trial. While NT-proBNP remains an appropriate biomarker for HFpEF with hypertension when used for diagnostic enrichment and serial group-level assessment over longer intervals, as in PARAGON-HF, EMPEROR-Preserved, DELIVER, and the 2025 Circulation guidance.^18–21^ Changes occurring during short-interval (<2–3 weeks) such as the time periods used in this trial cannot be interpreted because of biological and analytical variation.^18,22–24^ That being said, a larger trial in a similar patient population is currently being designed by us, which includes a longer treatment period (at least 3 months). NT-BNP levels will be tracked in this upcoming trial, for the reasons mentioned above.

Finally, BP measurements were made twice per day and were based on a single BP cuff measurement. From a practical standpoint, this was the best that could be achieved in a 6-week study performed in a patient’s home environment but also has the advantage of being taken in a familiar and relatively stress-free environment. Indeed, landmark studies in hypertension therapy have been published utilizing measurements performed in environments familiar to patients, such as a neighbourhood barbershop.^25^ Future studies will determine the optimal methodology and frequency of BP measurement to optimize the effect demonstrated in this study.

## Declarations

### Data Availability

No data were generated or analysed for this manuscript.

### Pre-registered Clinical Trial Number

None supplied.
